# Interpreting the interphase gap effect on the electrically evoked compound action potential

**DOI:** 10.1121/10.0009383

**Published:** 2022-02-04

**Authors:** Yi Yuan, Jeffrey Skidmore, Shuman He

**Affiliations:** 1 Department of Otolaryngology–Head and Neck Surgery, The Ohio State University, Columbus, Ohio 43212, USA; 2 Department of Audiology, Nationwide Children's Hospital, Columbus, Ohio 43205, USA yi.yuan@osumc.edu, Jeffrey.Skidmore@osumc.edu, Shuman.He@osumc.edu

## Abstract

This study demonstrated the effects of using different quantification methods and parameter scales on the sensitivity of the electrically evoked compound action potential (eCAP) to changes in the interphase gap (IPG). The IPG effect measured in two groups of cochlear implant (CI) users with different cochlear nerve (CN) health on seven eCAP measures was quantified using an absolute and a proportional difference method. The IPG effect provides an indicator for the functional status of the CN in human CI users. Specifying how the IPG effect is quantified is critical for accurate result interpretation.

## 1. Introduction

The number of surviving neural elements in the cochlear nerve (CN) and their responsiveness to electrical stimulation (i.e., functional status of the CN) has been shown to be important for cochlear implant (CI) outcomes (Pfingst *et al.*, 2015; Pfingst *et al.*, 2017; Schvartz-Leyzac *et al.*, 2020b; Schvartz-Leyzac and Pfingst, 2018; Skidmore *et al.*, 2020; Zhou and Pfingst, 2014). The sensitivity of the electrically evoked compound action potential (eCAP) to changes in the interphase gap (IPG) (i.e., the IPG effect) is associated with the survival of spiral ganglion neurons (SGNs) in guinea pigs (Prado-Guitierrez *et al.*, 2006; Ramekers *et al.*, 2014; Ramekers *et al.*, 2015; Schvartz-Leyzac *et al.*, 2019), and with the presumed functional status of the CN in human CI users (He *et al.*, 2018; He *et al.*, 2020a; Hughes *et al.*, 2018; Kim *et al.*, 2010; Schvartz-Leyzac and Pfingst, 2016, 2018; Skidmore and He, 2021).

Studies investigating the IPG effect on the eCAP in animal models showed different results from those measured in human CI users. Specifically, guinea pigs with lower densities of SGNs showed (1) larger IPG effects on the eCAP threshold, the maximum eCAP amplitude and the N1 latency, (2) smaller IPG effects on the slope of the eCAP input/output (I/O) function, and (3) smaller stimulation level offsets (Prado-Guitierrez *et al.*, 2006; Ramekers *et al.*, 2014; Ramekers *et al.*, 2015). In contrast, we recently found a different trend in human CI users when comparing the IPG effect between children with cochlear nerve deficiency (CND, i.e., a small or absent CN in imaging results) and children with normal-sized CNs (NSCNs) (He *et al.*, 2020a; Skidmore and He, 2021). Specifically, compared with children with NSCNs, children with CND tended to have (1) larger IPG effects on the maximum eCAP amplitude and the slope of the eCAP I/O function, (2) smaller IPG effects on the eCAP threshold and the N1 latency, and (3) larger stimulation level offsets (He *et al.*, 2020a; Skidmore and He, 2021).

In addition to differences in species, electrode placement, and duration of deafness (Hughes *et al.*, 2018; He *et al.*, 2020b), the difference in how the IPG effect is quantified across studies might be another important factor accounting for the discrepancy in results reported between animal models and human CI users (Ramekers *et al.*, 2014; He *et al.*, 2020a). Specifically, the IPG effect on the eCAP has been quantified using two methods: the absolute difference method and the proportional difference method. In the absolute difference method, the IPG effect is defined as the difference in eCAP results recorded at different IPGs. This method has been used to quantify the IPG effect on the slope of eCAP I/O function, the maximum eCAP amplitude, and the N1 latency (Ramekers *et al.*, 2014; Schvartz-Leyzac *et al.*, 2020a), as well as the stimulation level offset (Prado-Guitierrez *et al.*, 2006; Kim *et al.*, 2010; Ramekers *et al.*, 2014; Skidmore and He, 2021). In the proportional difference method, the IPG effect on the eCAP is defined as the proportional change relative to the eCAP result measured at one selected IPG. This method has been used to quantify the IPG effect on the slope of eCAP I/O function, the maximum eCAP amplitude, the eCAP threshold, and the N1 latency (Schvartz-Leyzac and Pfingst, 2016; He *et al.*, 2020a).

In addition to the quantification method, the choice of parameter scale (i.e., linear versus logarithmic scale) has been proposed to be important for assessing the IPG effect on the slope of the eCAP I/O function (Brochier *et al.*, 2021). However, this concept has not been verified on other eCAP measurements. This study aimed to demonstrate the importance of the choice of quantification method and parameter scale when reporting the IPG effect.

## 2. Materials and methods

This study reanalyzed eCAP data collected in He *et al.* (2020a). The eCAP parameters [i.e., dependent variables (DVs)] evaluated in this study included the eCAP threshold quantified using linear scaling units [i.e., nanocoulombs (nC)], the eCAP threshold quantified using logarithmic scaling units [i.e., clinical current level (CL)], the maximum slope of the eCAP I/O function estimated using the window method (Skidmore *et al.*, 2021) in a logarithmic input scale (*μ*V/dB), the overall slope of the eCAP I/O function estimated using linear regression (Abbas *et al.*, 1999; Kim *et al.*, 2010) in a linear scale (*μ*V/nC), the maximum eCAP amplitude, the N1 latency of the eCAP recorded at the maximum stimulation level, and the stimulation level offset. Outliers when calculating the slope of the I/O function or stimulation level offset were determined based on the goodness of fit (i.e., R^2^) using the three scaled median absolute deviation criterion (Leys *et al.*, 2013) and removed from further analysis. No outliers were removed for the eCAP threshold, the maximum eCAP amplitude, or the N1 latency.

The IPGs tested in this study included 7 and 42 *μ*s. For the absolute difference method, the IPG effect was quantified using the formula:

Absolute IPG effect size=DV measured for 42 μs IPG−DV measured for 7 μs IPG (1)

For the proportional difference method, the IPG effect was quantified using the formula:

$$\mathrm{Proportional}\;\mathrm{IPG}\;\mathrm{effect}\;\mathrm{size}=\frac{\mathrm{Absolute}\;\mathrm{IPG}\;\mathrm{effect}\;\mathrm{size}}{DV\;\mathrm{measured}\;\mathrm{for}\;\mathrm{the}\;7\;\mu s\;\mathrm{IPG}}$$ (2)

The group differences were compared using two-tailed, paired-samples t-tests. All data and statistical analyses were performed using MATLAB (v2019b, Mathworks Inc., Natick, MA).

## 3. Results

### 3.1 IPG effect: Absolute difference

**Fig. 1.**
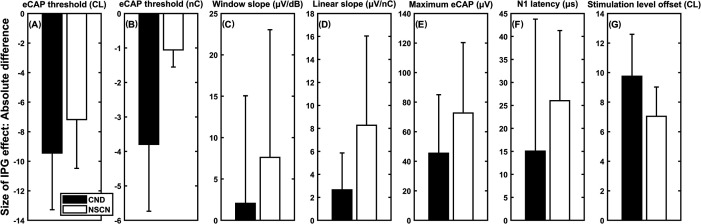
The means and SDs of the interphase gap (IPG) effect size on the seven dependent variables (DVs) calculated as the absolute difference in DVs measured at different IPGs. Results measured in children with cochlear nerve deficiency (CND) and children with normal-sized cochlear nerves (NSCNs) are indicated using filled bars and open bars, respectively.

Figure 1 depicts the size of the IPG effect on the seven DVs measured for both subject groups using the absolute difference method. Each panel shows the comparison of the means and standard deviations (SDs) measured for each DV. These results clearly show a group difference for each DV, which was confirmed by results of statistical analysis (Table 1). When increasing the IPG, children with CND had larger changes in the eCAP threshold (Panels A and B) and the stimulation level offset (Panel G), and smaller changes in the slope of the eCAP I/O function (Panels C and D), the maximum eCAP amplitude (Panel E), and the N1 latency (Panel F) than children with NSCNs. Similar trends in group differences were observed when using different parameter scales and slope-fitting methods (Panels A and B, Panels C and D).

**Table 1.** Results from paired sample t-tests for each dependent variable.

Dependent variable	Absolute difference	Proportional difference
eCAP threshold (CL)	t_(89)_ = −4.96, p < 0.001	t_(89)_ = 1.69, p = 0.094
eCAP threshold (nC)	t_(89)_ = −13.33, p < 0.001	t_(89)_ = −4.83, p < 0.001
Window slope (*μ*V/dB)	t_(63)_ = −2.49, p = 0.015	t_(63)_ = −0.34, p = 0.731
Linear slope (*μ*V/nC)	t_(66)_ = −5.05, p < 0.001	t_(66)_ = 2.76, p = 0.008
Maximum eCAP (*μ*V)	t_(89)_ = −3.94, p < 0.001	t_(89)_ = 3.11, p = 0.003
N1 latency (*μ*s)	t_(89)_ = −3.09, p = 0.003	t_(89)_ = −4.43, p < 0.001
Stimulation level offset (CL)	t_(68)_ = 6.70, p < 0.001	t_(68)_ = −0.50, p = 0.620

### 3.2 IPG effect: Proportional difference

**Fig. 2.**
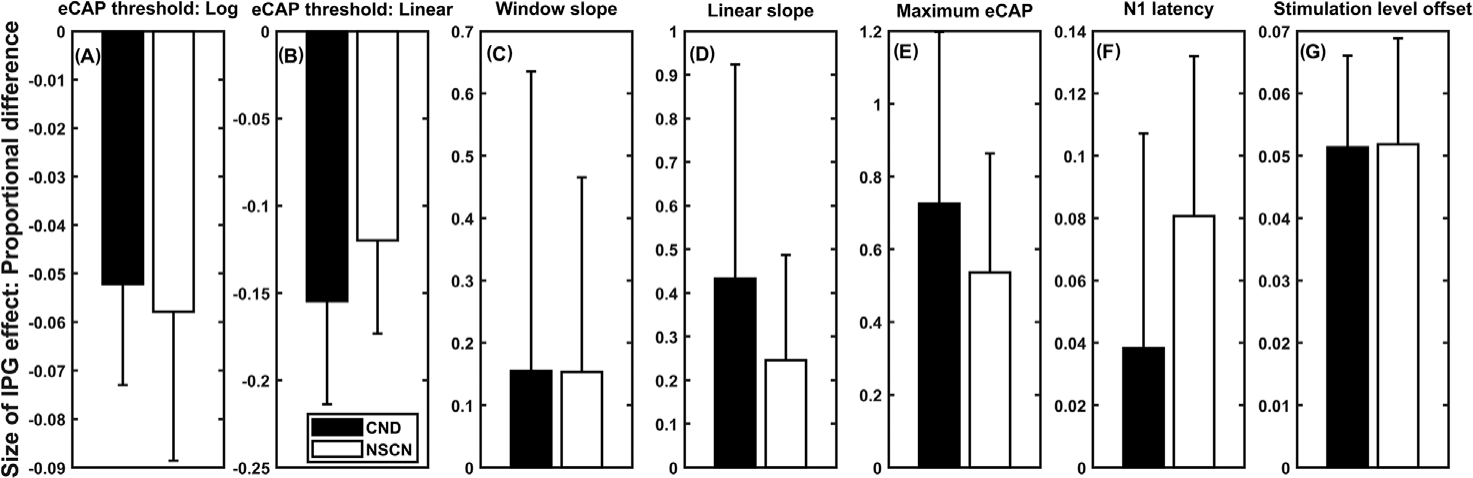
The means and SDs of the interphase gap (IPG) effect size on the seven dependent variables calculated with the proportional difference method. Results measured in children with cochlear nerve deficiency (CND) and children with normal-sized cochlear nerves (NSCNs) are indicated using filled bars and open bars, respectively.

Figure 2 shows means and SDs of the IPG effect size on the seven DVs measured using the proportional difference method for both subject groups. Compared to children with NSCNs, children with CND showed (1) significantly larger changes in the eCAP threshold calculated on a linear scale (Panel B), the overall slope of the eCAP I/O function calculated with linear regression (Panel D) and the maximum eCAP amplitude (Panel E), and (2) smaller changes in the N1 latency (Panel F) at longer IPGs. There was no statistically significant difference between the two groups for the eCAP threshold calculated on a logarithmic scale (Panel A), the maximum slope of the eCAP function calculated with the window method (Panel C), or the stimulation level offset (Panel G). The results of statistical analyses for each DV are provided in Table 1.

## 4. Discussion

The primary goal of this study was to investigate how different analysis methods affect the interpretation of the IPG effect. The present study showed that the group difference in IPG effect size on the eCAP could be greatly influenced by the quantification method and scale selection.

### 4.1 Quantification method

The difference in magnitude and direction of the group difference in IPG effect size was highly dependent on the eCAP parameter and quantification method. Specifically, the group difference changed direction for the linear slope and the maximum eCAP amplitude when changing from the absolute difference (similar trend as Schvartz-Leyzac *et al.*, 2020a) to the proportional difference (same trend as He *et al.*, 2020a). The group difference went from being significant to non-significant for the eCAP threshold calculated in a logarithmic scale, the slope calculated using the window method, and the stimulation level offset. Finally, the group difference was the same direction and remained statistically significant for the N1 latency and the eCAP threshold calculated in a linear scale.

### 4.2 Scale selection

Results of this study also showed that both the magnitude and the direction of group difference in IPG effect size could be dependent on the scale in which the eCAP threshold was calculated. Specifically, the relative group difference in IPG effect size was much larger when the eCAP threshold was calculated on a linear scale than on a logarithmic scale when using the absolute difference method [Figs. 1(A) and 1(B), respectively]. When using the proportional difference method, children with CND showed relatively smaller IPG effects on the eCAP threshold calculated on a logarithmic scale [Fig. 2(A)], but larger IPG effects on the eCAP threshold calculated on a linear scale [Fig. 2(B)].

The effect of method and scale on estimating the slope of the eCAP I/O function has been detailed in Skidmore *et al.* (2021), and therefore, is not discussed in this brief report.

### 4.3 Study limitations

It should be pointed out that some non-neural factors (e.g., electrode placement, impedance, etc.) can affect the IPG effect calculated using the absolute difference method on the slope of eCAP I/O function, the maximum eCAP amplitude, and the N1 latency. These non-neural factors should not affect the IPG effect calculated using the proportional difference method on these eCAP parameters. Unfortunately, we do not have sufficient data to fully assess the impact of these non-neural factors on the difference in IPG effects quantified used these two methods, which is a potential limitation of this study.

Due to the retrospective nature of this study and the inherent limitation of working with clinical patient populations, we are unable to unravel the neurophysiological mechanisms underlying the complicated data pattern reported in this study, which is another potential limitation. Further studies are definitely warranted.

## 5. Conclusion

Results of this study revealed that the method for quantifying the IPG effect size and the choice of parameter scale impact the results and their interpretations of the IPG effect on the eCAP between children with CND and children with NSCNs. Robust group differences in the IPG effect on several eCAP parameters are still observed when different analyzing techniques are applied, which further supports the idea that eCAP sensitivity to changes in IPG can provide an indicator for the functional status of the CN in human CI users. However, a careful selection of eCAP parameters and specifying how the IPG effect is quantified are critical for accurate result calculation and interpretation for any studies that include the IPG effect as part of the testing paradigms.
